# The Seamen's Hospital

**Published:** 1915-06-12

**Authors:** 


### The Seamen's Hospital.

THE following letter from Lord Devonport, relating to the Seamen's Hospital, Greenwich, appeared in the *Times* of June 2 :—

Sir,—I have been requested to act as chairman of a Special Appeal Committee on behalf of the Seamen's Hospital at Greenwich, and I beg to be permitted to direct attention through your columns to the special needs of the institution at this moment. The Seamen's Hospital has cared for the sick and injured merchant seamen of the Empire for nearly a century; practically every port in the British Islands, every quarter of the globe, contributes from time to time patients, and benefits by its humane work.

Since the war large numbers of wounded soldiers and sailors have been received and tended, and this increased strain, so willingly undertaken, makes the appeal even more urgent.

The efficiency of this hospital of 300 beds is actually crippled by the delay of urgently needed improvements occasioned by a lack of funds. There are no isolation wards, and the *x*-ray and electric department, now of such supreme importance, is out of date. For want of adequate accommodation the nurses are obliged to sleep five in one room, and have no separate quarters for quiet and rest in their off-duty hours, and last, but not least, there are no lifts throughout the hospital, and much unnecessary suffering is caused in consequence, the patients having to be carried up and down steep stairs in course of treatment, embracing conveyance to and from the operating theatre. The growth of the Port of London and its absolutely certain increase with the termination of the war, the extension of the Empire's oversea trade makes it imperatively essential that the hospital should be able to cope with ever-gathering demands.

The space, the opportunity, the organisation is there, but only by a considerable capital expenditure can sufficiency and efficiency of accommodation and equipment be afforded. These are not ordinary times for the men of our mercantile marine; we have every reason to be proud of their courage, of their example of devotion to duty, as well as their record of invaluable co-operation with the Fleet since war began. In whatever capacity, whether serving on Fleet auxiliaries, in the transport service or mine-sweepers, on patrol duty, or in following the ordinary avocations of the merchant service, these brave seamen mostly work unnoticed and without hope of reward, carrying, as we daily realise, their lives in their hands. I am sanguine enough to hope that this appeal will afford a welcome opportunity to all of us gratefully to recognise a debt we owe to a very gallant class.

I shall be glad to receive contributions in aid of the Appeal Fund at 41 Grosvenor Place, S.W.—Yours faithfully, DEVONPORT.

Port of London Authority, E.C., June 1.

